# Fibrinopeptide A Family Biomarker Identification at Single Molecule Level

**DOI:** 10.1021/acscentsci.3c00088

**Published:** 2023-02-03

**Authors:** Sebastien Balme

**Affiliations:** Institut Européen des Membranes, UMR5635 UM ENCSM CNRS, Place Eugène Bataillon, 34095 cedex 5 Montpellier, France

T he detection and analysis of peptide biomarkers is one of the most important tools for the early diagnosis of many diseases, which is a key element for personalized medicine. One of the main challenges in this field is the ability to identify small sequence modifications (length, phosphorylation, post-translational modification, and mutation) that are often associated with a given pathology. In this issue of *ACS Central Science*, Pelta, Cressiot, and co-workers report a breakthrough approach to detect and discriminate fibrinopeptide A (FPA) family biomarkers at the single molecule level.^1^ Unphosphorylated and phosphorylated FPA are clinical biomarkers of the coagulation system, whose concentration in blood increases in the case of heart disease.^2^ In addition, the N-terminal cleaved derivatives FPA-3 and FPA-6 are biomarkers of cancer.^3^ These make this peptide a target of choice for the development of a point-of-care device as soon as a simple, accurate, and low-cost detection and analysis technique can be used.

Since its proof of concept 30 years ago,^4^ nanopore technology has allowed considerable advances in biomolecule analysis. This technology is based on analysis of the current perturbation induced by the passage of a macromolecule inside a biological nanopore (a-hemolysin, aerolysin, etc.). It has the advantages of being inexpensive, accurate, and extremely reproducible, and has thus allowed the development of long-read DNA sequencing.^5^ Following this, the analysis of protein and peptide sequences or their modifications is likely the most important challenge in the field of nanopore sensing.^6^ Major recent advances in this field include homopolypeptide identification,^7^ protein fragment identification after enzymatic degradation,^8^ peptide reading at single amino-acid resolution,^9^ and detection of familial mutation of Aβ peptide.^10^

**Figure 1:**
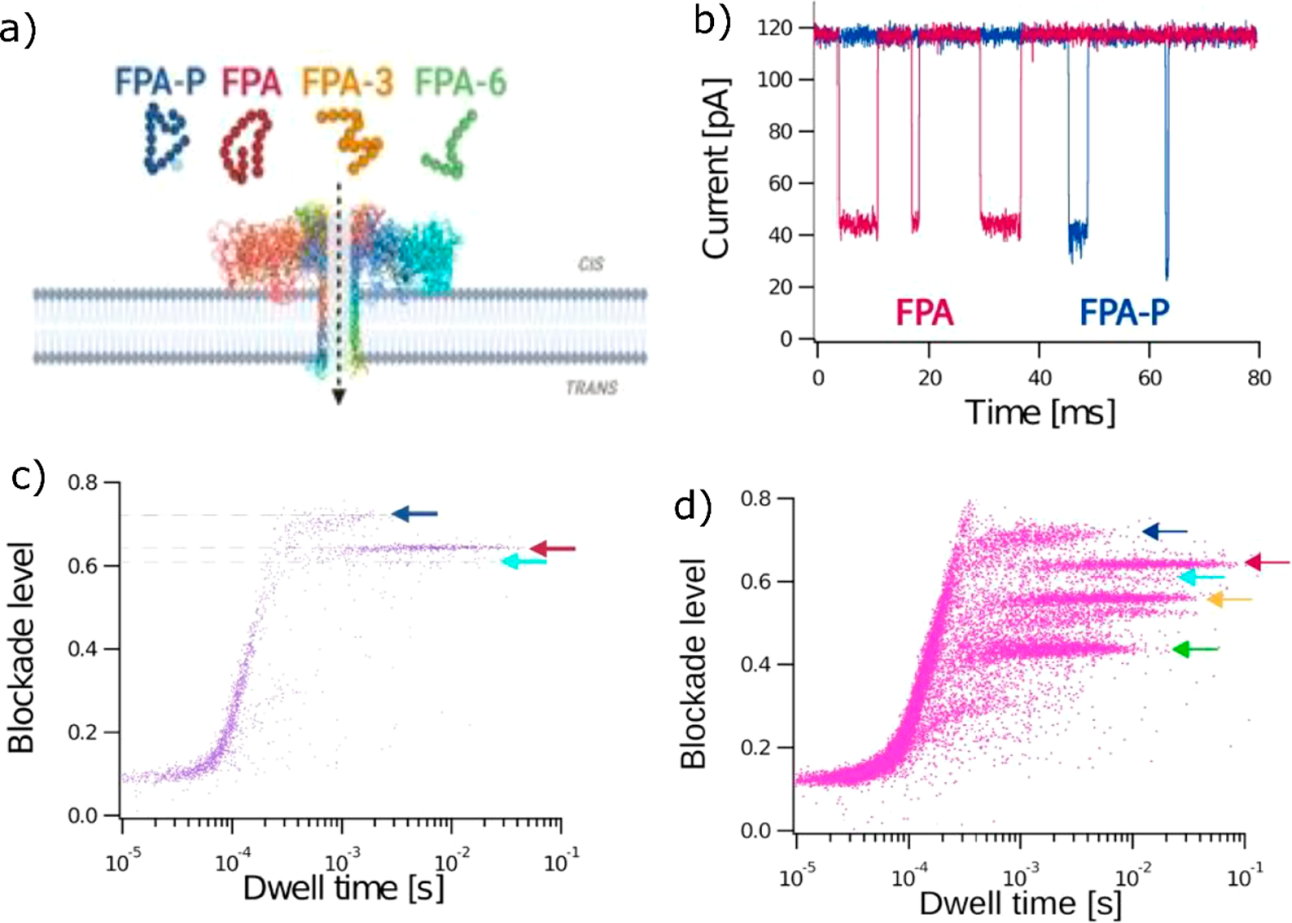
Scheme of FPA detection using the aerolysin nanopore. (b) Representative current traces recorded over 80 ms for FPA (red) and FPA-P (blue) showing the difference in the current blockade amplitude. (c) Scatter plots showing normalized blockade level, defined as (I0 – Ib)/I0, against the dwell time for the mixture of FPA and FPA-P. The arrows denote the different populations of FPA (red) and FPA-P (blue). (D) Scatter plots for the mixture of FPA, FPA-P, FPA-3, and FPA-6 and their associate population denoted by the red, blue, yellow, and green arrows, respectively. Reproduced with permission from ref (1). Copyright 2023. The Authors. Published by American Chemical Society.

Pelta, Cressiot, and co-workers go further in the application of biological nanopores for the sensing of peptide biomarkers, aiming to discriminate FPA, its phosphorylated form (FPA-P), and two cleaved forms (FPA-3 and FPA-6). The team demonstrated that the amplitude of the current blockage is greater for phosphorylated than unphosphorylated FPA (Figure 1). The significant difference in the current blockade level allowed the authors to discriminate the two forms from a mixture by eye without the need for further statistical analysis or extensive signal processing. In addition, two discrete blocking levels were shown for the phosphorylated form relating two conformational states that could significantly improve the accuracy of the nanopore sensor. Using the same method, the team analyzed the truncations FPA-3 and FPA-6, showing as expected a decrease of the blocking amplitude with the size of the peptide (Figure 1). Here again, the precision of the nanopore allows a visual discrimination of the biomarker mixture by plotting the histogram of the current blocking amplitudes, where each molecule induces a discrete level in the plot.
